# Community acquired vs hospital acquired acute kidney injury. mortality and timing of renal replacement therapy

**DOI:** 10.1186/2197-425X-3-S1-A257

**Published:** 2015-10-01

**Authors:** G Moreno-Gonzalez, X Perez-Fernandez, P Cardenas-Campos, N Betancur-Zambrano, V Gumucio, D Toapanta, S Contreras, V Corral-Velez, J Sabater, SIRAKI Group

**Affiliations:** Hospital Universitari de Bellvitge, Intensive Care, Barcelona, Spain

## Objectives

To analyse differences in survival between patients presenting community acquired advanced acute kidney injury (CAKIN3) and hospital acquired advanced acute kidney injury (HAKIN3). We also evaluated Renal Replacement Therapy (RRT) timing impact in these two populations.

## Methods

We studied a cohort of 217 critically ill patients all of them presenting advanced acute kidney injury (AKIN3) at some point of their hospital stay. Patients with previous advanced chronic kidney disease (CKD5) were excluded. CAKIN3 was defined as those patients who presented AKIN3 at hospital admission and HAKIN3 was defined as those patients who presented no AKI at hospital admission. Both groups studied presented AKIN3 within the first 24h of ICU admission. To evaluate RRT timing impact we defined as Early RRT those patients whom were initiated on RRT within the first 48 hours from hospital admission (CAKIN3) or within the first 48 hours from ICU admission (HAKIN3). Patients with more than 7 days from hospital admission (CAKIN3) or ICU admission (KAKIN3) to RRT initiation were both excluded from RRT timing analysis. Non RRT control group was also included in timing analysis.

## Results

90 days mortality was 51.5% in CAKIN3 patients and 71.1 % in HAKIN3 patients (p = 0.005). RRT was required in 82.8% of CAKIN3 patients vs 89.2% of HAKIN3 patients.

No differences between CAKIN3 and HAKIN3 were observed in terms of age (62 ± 15 vs 61 ± 14 years), SOFA score (12 ± 4 vs 12 ± 4), baseline creatinine (100 ± 46 vs 108 ± 50 umol/L), Lactate at ICU admission (6.2 ± 7 vs 5.2 ± 5.5 mmol/L), days from ICU to RRT (2.5 ± 6.8 vs 1.5 ± 1.8 days), urine output at RRT initiation (0.25 ± 0.34 vs 0.20 ± 0.23 ml/kg/h), mechanical ventilation at RRT initiation (81% vs 79%), or presence of shock at RRT initiation (94.6% vs 90.2%).

Differences between CAKIN3 and HAKIN3 were observed in terms of creatinine at hospital admission (379 ± 268 vs 108 ± 54 umol/L), creatinine at ICU admission (372 ± 225 vs 260 ± 134 umol/L), urine output within the first 24h of ICU admission (0.40 ± 0.49 vs 0.28 ± 0.30 ml/kg/h), creatinine at RRT initiation (435 ± 205 vs 337 ± 128 umol/L), pH at RRT initiation (7.25 ± 0.13 vs 7.29 ± 0.11), diabetes mellitus (31.5% vs 19%), and Chronic obstructive pulmonary disease-COPD (23.1% vs 9.6%). Most of CAKIN3 patients were medical (60.3%) and most of HAKIN3 patients were surgical (58.3%).

In CAKIN3 patients Early RRT improved 90 day survival compared to Late RRT and non RRT patients (p = 0.049). In HAKIN3 patients Early RRT had no survival impact compared to Late RRT or non-RRT patients.

## Conclusions

Community acquired advanced AKI seems to present better outcome respect to hospital acquired advanced AKI. Early RRT seems to improve outcome in community acquired advanced AKI but has no beneficial impact on hospital acquired advanced AKI.

